# The Urokinase Receptor (uPAR) Facilitates Clearance of *Borrelia burgdorferi*

**DOI:** 10.1371/journal.ppat.1000447

**Published:** 2009-05-22

**Authors:** Joppe W. R. Hovius, Maarten F. Bijlsma, Gerritje J. W. van der Windt, W. Joost Wiersinga, Bastiaan J. D. Boukens, Jeroen Coumou, Anneke Oei, Regina de Beer, Alex F. de Vos, Cornelis van 't Veer, Alje P. van Dam, Penghua Wang, Erol Fikrig, Marcel M. Levi, Joris J. T. H. Roelofs, Tom van der Poll

**Affiliations:** 1 Center for Experimental and Molecular Medicine (CEMM), Academic Medical Center, University of Amsterdam, AMC, Amsterdam, The Netherlands; 2 Center for Infection and Immunity Amsterdam (CINIMA), Academic Medical Center, University of Amsterdam, AMC, Amsterdam, The Netherlands; 3 Department of Medicine, Academic Medical Center, University of Amsterdam, AMC, Amsterdam, The Netherlands; 4 Heart Failure Research Center, Academic Medical Center, University of Amsterdam, AMC, Amsterdam, The Netherlands; 5 Department of Medical Microbiology, Academic Medical Center, University of Amsterdam, AMC, Amsterdam, The Netherlands; 6 Onze Lieve Vrouwe Gasthuis, Department of Medical Microbiology, Amsterdam, The Netherlands; 7 Yale University, School of Medicine, Section of Infectious Diseases, Department of Internal Medicine, New Haven, Connecticut, United States of America; 8 Department of Pathology, Academic Medical Center, University of Amsterdam, AMC, Amsterdam, The Netherlands; Tufts University School of Medicine, United States of America

## Abstract

The causative agent of Lyme borreliosis, the spirochete *Borrelia burgdorferi*, has been shown to induce expression of the urokinase receptor (uPAR); however, the role of uPAR in the immune response against *Borrelia* has never been investigated. uPAR not only acts as a proteinase receptor, but can also, dependently or independently of ligation to uPA, directly affect leukocyte function. We here demonstrate that uPAR is upregulated on murine and human leukocytes upon exposure to *B. burgdorferi* both in vitro as well as in vivo. Notably, *B. burgdorferi*-inoculated C57BL/6 uPAR knock-out mice harbored significantly higher *Borrelia* numbers compared to WT controls. This was associated with impaired phagocytotic capacity of *B. burgdorferi* by uPAR knock-out leukocytes in vitro. *B. burgdorferi* numbers in vivo, and phagocytotic capacity in vitro, were unaltered in uPA, tPA (low fibrinolytic activity) and PAI-1 (high fibrinolytic activity) knock-out mice compared to WT controls. Strikingly, in uPAR knock-out mice partially backcrossed to a *B. burgdorferi* susceptible C3H/HeN background, higher *B. burgdorferi* numbers were associated with more severe carditis and increased local TLR2 and IL-1β mRNA expression. In conclusion, in *B. burgdorferi* infection, uPAR is required for phagocytosis and adequate eradication of the spirochete from the heart by a mechanism that is independent of binding of uPAR to uPA or its role in the fibrinolytic system.

## Author Summary

Lyme borreliosis is caused by the spirochete *Borrelia burgdorferi* and is transmitted through ticks. Since its discovery approximately 30 years ago it has become the most important vector-borne disease in the Western world. The pathogenesis of this complex zoonosis is still not entirely understood. We here demonstrate that the urokinase receptor (uPAR) is upregulated in mice and humans upon exposure to *B. burgdorferi* in vitro and in vivo. Importantly, we describe the function of uPAR in the immune response against the spirochete; using uPAR knock-out mice, we show that uPAR plays an important role in phagocytosis of *B. burgdorferi* by leukocytes both in vitro as well as in vivo. In addition, we show that the mechanism by which uPAR is involved in the phagocytosis of *B. burgdorferi* is independent of ligation to its natural ligand uPA or uPAR's role in fibrinolysis. Our study contributes to the understanding of the pathogenesis of Lyme borreliosis and might contribute to the development of innovative novel treatment strategies for Lyme borreliosis.

## Introduction

Lyme borreliosis, an emerging tick-borne disease in both the New and Old world, is caused by spirochetes belonging to the *Borrelia burgdorferi* sensu lato group and is predominantly transmitted by *Ixodes* ticks [1]. In the United States *Borrelia burgdorferi* sensu stricto, from here on referred to as *B. burgdorferi*, is the only prevalent *Borrelia* species, whereas in Europe three *Borrelia* species - *B. burgdorferi*, *Borrelia garinii* and *Borrelia afzelii* – are able to cause Lyme borreliosis [2],[3]. In humans, all three species frequently cause an erythematous cutaneous lesion, *erythema migrans*. In later stages of infection spirochetes can disseminate and cause disease that affects the joints, cardiac conduction system, central nervous system and the skin [4].

*Borrelia* has been shown to differentially express specific genes to inhibit, modulate or to bypass the host immune system [5] and to bind to host molecules in order to establish a persisting infection. In addition, *B. burgdorferi* can interact with the host fibrinolytic system [6]. *B. burgdorferi* abuses host plasminogen activators to activate plasminogen within the tick gut to facilitate migration through the arthropod vector [7]. However, plasminogen is not critical for transmission and infection, since plasminogen deficient mice do develop an infection after intradermal inoculation with *B. burgdorferi* [7]. In in vitro studies, the spirochete causes upregulation of the urokinase Plasminogen Activator (uPA) [8],[9], the Plasminogen Activator Inhibitors (PAI)-1 and 2 [10],[11], and the uPA Receptor (uPAR; CD87; PLAUR) [12],[13]. uPAR is a multi-ligand receptor with a high affinity for uPA, but also vitronectin, many integrins and G-protein-coupled receptors, and is expressed by many different cell types, including leukocytes [14]. Binding of uPA to uPAR results in formation of plasmin at the leading edge of cells facilitating leukocyte migration by pericellular proteolysis of extracellular matrix proteins [14]. Besides functioning as a proteinase receptor, uPAR also affects leukocyte migration and adhesion [15]–[20], as well as phagocytosis [19],[21], through intracellular signaling. This occurs, in part, independently of ligation of uPA by uPAR [22],[23].

Importantly, uPAR has been shown to contribute to activation and mobilization of leukocytes in bacterial infections [14],[15],[19]–[24]. To elucidate the role and function of uPAR in the development of Lyme borreliosis in vivo we infected wildtype (WT) and uPAR knock-out C57BL/6 mice with *B. burgdorferi* sensu stricto and monitored *B. burgdorferi* numbers in multiple organs, histopathological changes of tibiotarsi and heart, and host immune responses. In addition, to investigate whether the observed phenotype in uPAR knock-out C57BL/6 mice was dependent on uPAR's role in the fibrinolytic system or dependent on the interaction with uPA we also investigated the course of Lyme borreliosis in tPA, PAI-1 and uPA knock-out C57BL/6 mice. Moreover, we investigated the course of *Borrelia* infection in uPAR knock-out mice partially backcrossed to a C3H/HeN genetic background to assess the role of uPAR in mice more susceptible for infection with *B. burgdorferi*.

## Results

### *Borrelia burgdorferi* upregulates uPAR expression in mice and humans

**Figure 1:**
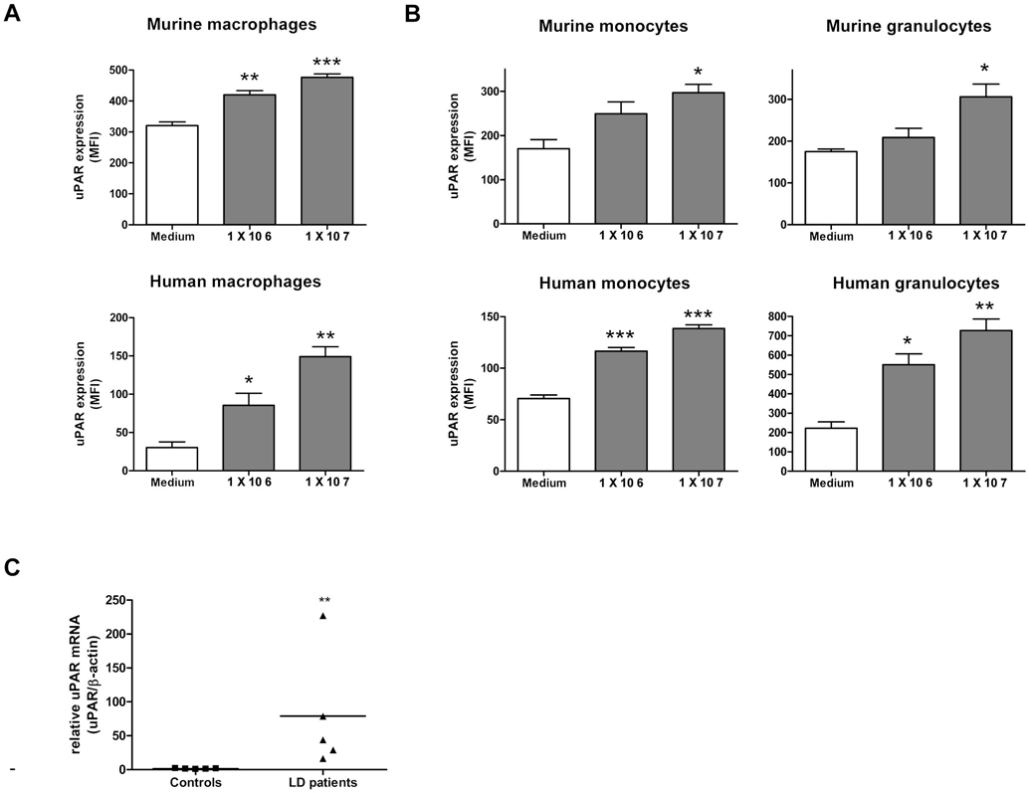
*Borrelia burgdorferi* induces upregulation of the urokinase receptor on murine and human leukocytes in vitro and in vivo. (A) Viable *B. burgdorferi* induces uPAR expression on murine and human macrophages. Murine peritoneal macrophages, and ex vivo generated human macrophages, (1×10^5^) were stimulated with viable *Borrelia burgdorferi* (strain B31) for 16 hours (Cell∶*B. burgdorferi* = 1∶10 or 1∶100). Cells were harvested and analyzed for uPAR expression by FACS analysis. (B) Viable *B. burgdorferi* induces uPAR expression on murine and human granulocytes and monocytes. Murine and human whole blood was incubated with viable *B. burgdorferi* for 16 hours. Erythrocytes were lysed and cells were co-stained for granulocytes or monocytes markers and uPAR and analyzed by FACS analysis. (C) Expression of uPAR is increased in skin biopsies from Lyme borreliosis patients. Total RNA was isolated from biopsies derived from culture and PCR confirmed *B. burgdorferi* positive erythema migrans lesions from Lyme borreliosis patients (n = 5) or healthy controls (n = 5) and subjected to quantitative uPAR and β-actin RT-PCR. We could not detect uPAR mRNA in healthy controls, for these samples the level of uPAR mRNA was set at the detection limit. Expression of uPAR mRNA was corrected for β-actin mRNA expression and depicted as a relative number. Expression of uPAR of one of the healthy controls was set at 1. Graphs in panels (A and B) are representative of at least three independent experiments and error bars represent the mean of triplicates within one experiment±SEM. A *p*-value<0.05 was considered statistically significant. * indicating *p*<0,05; ** *p*<0,01 and *** *p*<0,001.

Previous reports have shown that uPAR is upregulated on both a monocytic cell line and primary monocytes upon activation with *B. burgdorferi* [12],[13]. We here show that in vitro stimulation with different concentrations of viable *B. burgdorferi* resulted in significantly increased uPAR expression on both murine peritoneal macrophages and ex vivo generated – peripheral blood mononuclear cells-derived - human macrophages (**Figure 1A** and **Figure S1A**). In addition, using murine and human whole blood we observed similar results for granulocytes and monocytes (**Figure 1B** and **Figure S1B**). By contrast, non-phagocytotic cells, i.e. T lymphocytes, did not upregulate uPAR upon ex vivo exposure to *B. burgdorferi* (**Figure S1D**). Other *Borrelia* species, such as *B. garinii* strain PBi and *B. afzelii* strain pKo - both able to cause Lyme borreliosis - also induced enhanced uPAR expression on leukocytes (data not shown). To determine whether uPAR is upregulated in humans upon *B. burgdorferi* infection, we quantified uPAR expression in transcutaneous skin biopsies from *B. burgdorferi* PCR and culture confirmed positive erythema migrans patients and healthy controls. We could not detect uPAR expression in control patients, where as we could easily detect uPAR expression in the diseased group (**Figure 1C**). Lastly, in WT C57BL/6 mice inoculated intraperitoneally with viable *B. burgdorferi* for 1 hour we observed a significant upregulation of uPAR on the surface of (F4/80 positive) macrophages (**Figure S1C**).

### C57BL/6 uPAR knock-out mice exhibit increased *B. burgdorferi* numbers in vivo and impaired phagocytosis of *B. burgdorferi* in vitro

**Figure 2:**
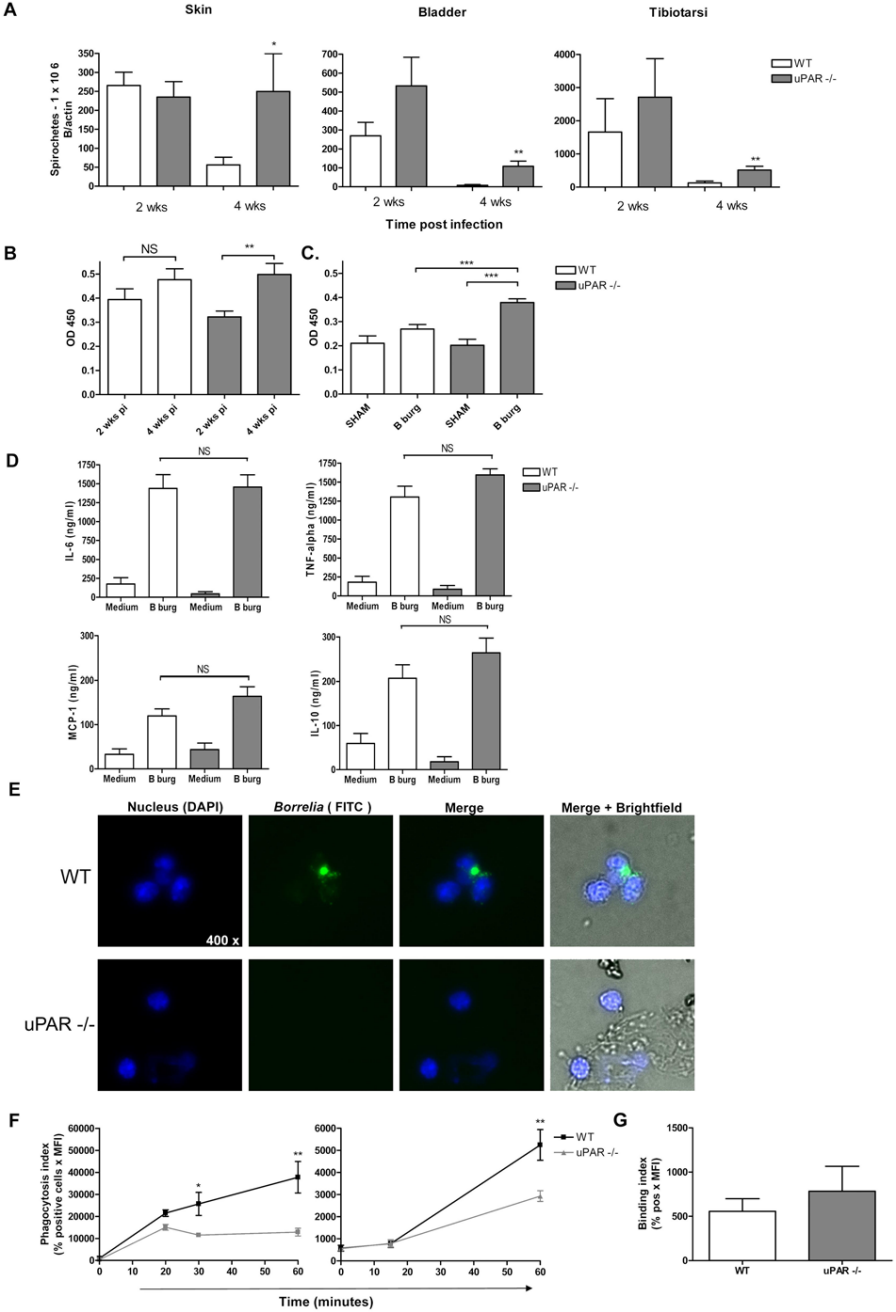
The urokinase receptor (uPAR) is involved in clearance of *B. burgdorferi*. (A) Urokinase receptor knock-out C57BL/6 mice display higher systemic *B. burgdorferi* numbers. WT and uPAR −/− mice were inoculated with *B. burgdorferi* and sacrificed two and four weeks post infection. DNA was extracted from the indicated tissues and subjected to quantitative *Borrelia flab* and mouse *β-actin* PCR. In sham inoculated mice (2 to 3 per group) we did not detect *B. burgdorferi* DNA. Six to eight mice per group were used and bars represent the mean±SEM. (B and C) Urokinase receptor knock-out C57BL/6 mice develop more rigorous IgG responses. Sera from C57BL/6 WT and uPAR knock-out mice, 2 and 4 weeks post *B. burgdorferi* (B burg) or sham inoculation (SHAM) was used for whole cell *B. burgdorferi* ELISA. Thus, we determined total IgG directed against *B. burgdorferi* (B) and IgG subclasses, of which only IgG1 (C) is shown. (D) WT and uPAR −/− macrophages produce similar levels of pro-inflammatory cytokines when exposed to viable *B. burgdorferi* in vitro. Peritoneal macrophages were stimulated with control medium (medium) or *B. burgdorferi* (B burg) for 16 hours. The supernatant was analyzed for cytokine production using a mouse inflammation cytometric bead array. (E and F) Urokinase receptor deficient granulocytes and macrophages are incapable of adequately phagocytosing *B. burgdorferi*. Whole blood or peritoneal macrophages were incubated with CFSE-labeled viable or heat-killed FITC-labeled *B. burgdorferi* at 37°C or at 4°C as a control. Phagocytosis was stopped by transferring the tubes to ice and extracellular bacteria were quenched by addition of a quenching dye containing Trypan blue. When whole blood was used erythrocytes were lysed before cells were DAPI stained and subjected to fluorescent microscopy (E) or stained for Gr-1 (granulocytes) and subjected to FACS analysis (F; left panel). Peritoneal macrophages were directly subjected to FACS analysis (F; right panel). Phagocytosis was depicted as the phagocytosis index [64],[65]: mean fluorescence intensity (MFI)×percentage (%) positive cells) at 37°C minus (MFI×% positive cells at 4°C). Six to eight mice per group were used, graphs represent the mean±SEM and are representative of three independent experiments. (G) *B. burgdorferi* binds equally well to WT and uPAR −/− macrophages. A similar experiment as described in (F) was performed, albeit at 4°C and without the addition of quenching dye to determine binding of *B. burgdorferi* to peritoneal macrophages. Binding is expressed as the binding index: % CFSE positive cells×MFI. Four to six mice per group were used and bars represent the mean±SEM. The experiment was repeated twice. A *p*-value<0.05 was considered statistically significant. * indicating *p*<0,05; ** *p*<0,01 and *** *p*<0,001.

**Figure 3:**
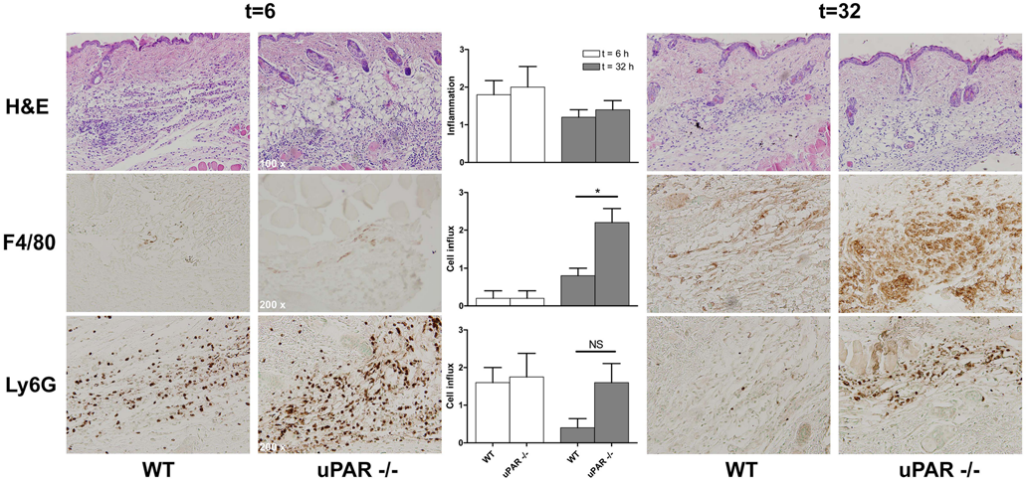
Leukocyte migration in uPAR knock-out mice in response to *B. burgdorferi* infection in vivo. C57BL/6 WT and uPAR knock-out mice were intradermally injected with 1×10^6^ *B. burgdorferi* in PBS in the midline of the neck and mice were sacrificed 6 or 32 hours post inoculation. Skin was harvested, formalin fixed and imbedded in paraffin. Five µm-thick sagittal skin sections were processed and H&E, Ly6G and F4/80 stained by routine histological techniques. Control animals injected with PBS alone did not display influx of leukocytes (data not shown). Slides were scored for influx of leukocytes by an independent pathologist who was blinded to the experimental design. Influx was semi-quantitatively scored on a scale from 0–3, with 0 being no, 1 mild, 2 moderate, and 3 being severe diffuse infiltration. Per group and time point 5 five mice were used, error bars represent SEM. Representative sections are depicted in the figure. A *p*-value<0.05 was considered statistically significant. * indicating *p*<0,05, i.e. p = 0,0259; NS, not significant, i.e. p = 0,1475.

To assess the role of uPAR in the immune response against *B. burgdorferi* vivo, we infected C57BL/6 WT and uPAR knock-out mice with *B. burgdorferi* and sacrificed mice two and four weeks post infection. By quantitative PCR we assessed *B. burgdorferi* numbers in skin, bladder and tibiotarsi post mortem. C57BL/6 uPAR deficient mice harbored higher *B. burgdorferi* numbers compared to WT animals in all tissues examined. This was most pronounced, and statistically significant, four weeks post infection (**Figure 2A**). These data were underscored by the fact that two weeks post infection only 3/8 bladder tissue cultures were positive in WT mice versus 7/7 in uPAR knock-out mice (Chi-square p = 0,026). We did not determine *B. burgdorferi* numbers in cardiac tissue in these experiments since the heart were used in toto for histopathology. In line with higher systemic *B. burgdorferi* numbers in uPAR deficient mice a significant increase in total IgG against *B. burgdorferi* over time (**Figure 2B**), and significantly higher IgG1 antibody levels four week post infection, were observed (**Figure 2C**). We detected no differences in IgM and IgG2b subclass-levels four weeks post infection (data not shown). To obtain a first insight into the mechanism by which uPAR deficiency could impact pathogen burden after infection with *B. burgdorferi* we stimulated leukocytes with viable spirochetes in vitro. We harvested peritoneal macrophages from C57BL/6 WT and uPAR knock-out mice, which we stimulated with viable *B. burgdorferi* (Cell∶*Borrelia* = 1∶50) for 16 hours. We demonstrate that *Borrelia* induced similar cytokine levels in WT and uPAR deficient macrophages (**Figure 2D**). We obtained comparable results when we stimulated whole blood in a similar fashion (data not shown). Next, because uPAR has been shown to play a crucial role in phagocytosis of *Escherichia coli* by neutrophils [19],[21],[22], we investigated whether WT and uPAR knock-out neutrophils and macrophages differed in their capacity to phagocytose *B. burgdorferi*. In these assays extracellular bacteria were quenched by addition of a quenching dye containing Trypan blue. We demonstrate that both uPAR knock-out neutrophils (in whole blood) and uPAR knock-out peritoneal macrophages were significantly less capable of phagocytosing *B. burgdorferi*, using either heat-killed FITC-labeled or viable CFSE-labeled *B. burgdorferi* (**Figure 2E** and **F** and **Figure S2**). Confocal microscopy confirmed labeled bacteria were localized intracellularly (**Figure S3A** and **B**). To distinguish between binding and phagocytosis we performed similar experiments, but at 4°C and without the addition of quenching solution. These experiments showed no difference in the capacity of WT and uPAR deficient leukocytes to bind *B. burgdorferi* (**Figure 2G**). In addition, binding experiments with recombinant human uPAR and viable *B. burgdorferi* failed to show direct binding of the spirochete to uPAR (data not shown). Since uPAR has been shown to be of importance in the migration of leukocytes, we also investigated whether there was impaired migration of leukocytes in *B. burgdorferi*-infected uPAR knock-out mice. We intradermally inoculated C57BL/6 WT and uPAR knock-out mice with *B. burgdorferi* or controls and harvested skin at 0, 6 or 32 hours post infection. We did not observe influx of immune cells at t = 0 (data not shown). By H&E, Ly6G and F4/80 stainings on sagittal skin sections we did observe an evident influx of immune cells and inflammation at t = 6 hours, however there were no differences between WT and uPAR knock-out mice (**Figure 3**). As has been shown by others [25], the predominant cells at this early time point were granulocytes (**Figure 3**). Importantly, these data show that the phenotype in uPAR knock-out mice is not explained by impaired influx of immune cells at the site of inoculation allowing for more dissemination of the spirochete. By contrast, later in the course of infection, at t = 32 hours, we observed a more pronounced influx of macrophages in uPAR knock-out mice compared to WT controls, which probably is explained by the increased *Borrelia* burden in uPAR knock-out mice (**Figure 3**). In conclusion, higher *B. burgdorferi* numbers in C57BL/6 uPAR knock-out mice compared to WT mice could be explained by a decreased phagocytotic capacity of uPAR deficient leukocytes observed in vitro, but not by impaired migration of uPAR deficient leukocytes.

### Higher *B. burgdorferi* numbers and impaired phagocytotic capacity in C57BL/6 uPAR knock-out mice are independent of ligation of uPA to uPAR

**Figure 4:**
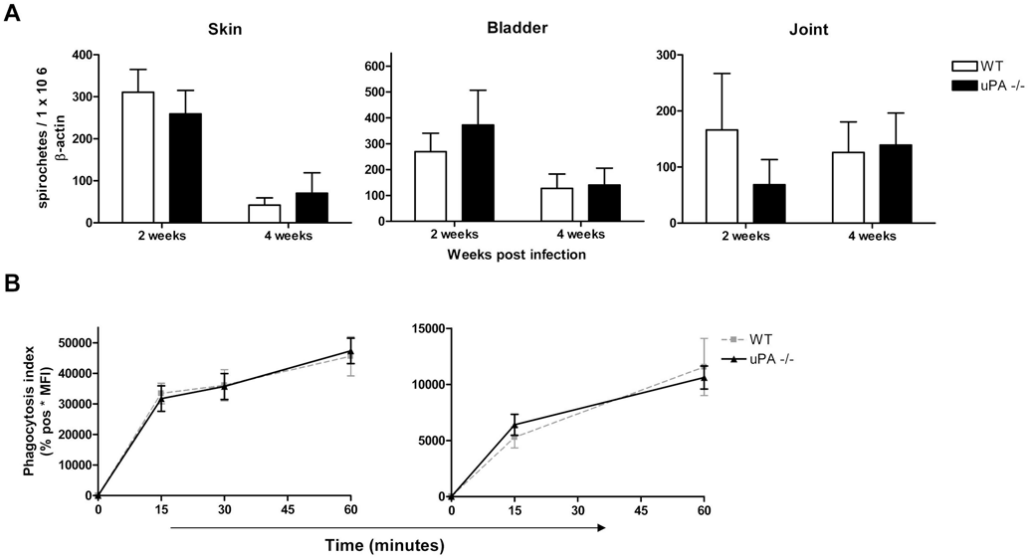
The urokinase activator (uPA) is not involved in clearance of the spirochete. (A) Urokinase activator knock-out C57BL/6 mice display similar systemic *B. burgdorferi* numbers compared to WT controls. WT and uPA −/− mice were inoculated with *B. burgdorferi* and sacrificed two and four weeks post infection. DNA was extracted from the indicated tissues and subjected to quantitative *Borrelia flab* and mouse *β-actin* PCR. Six to eight mice per group were used and *B. burgdorferi* numbers are depicted as described in Figure 2A. (B) Urokinase activator deficient granulocytes and macrophages (solid lines) are just as capable as WT controls (dotted lines) of phagocytosing *B. burgdorferi*. Phagocytosis assays were performed as described in Figure 2F. Six to eight mice per group were used, error bars represent SEM and the graphs are representative of two independent experiments. A *p*-value<0,05 was considered statistically significant.

Since uPAR has been suggested to affect function of leukocytes in both an uPA-dependent as well as an uPA-independent fashion we also assessed the course of *B. burgdorferi* infection in C57BL/6 uPA knock-out mice. Both 2 and 4 weeks post *B. burgdorferi* infection, C57BL/6 WT and uPA deficient mice displayed similar *Borrelia* numbers in all tissues examined as detected by quantitative PCR (**Figure 4A**). In addition, compared to WT controls, uPA deficient neutrophils and peritoneal macrophages were equally capable of phagocytosing *B. burgdorferi* (**Figure 4B**). These data suggest that the phenotype observed in C57BL/6 uPAR knock-out mice was independent of ligation of uPA to uPAR.

### Higher *B. burgdorferi* numbers and impaired phagocytotic capacity in C57BL/6 uPAR knock-out mice are independent of uPAR's role in the fibrinolytic system

Next, since uPAR has been shown to affect function of leukocytes through its role in the fibrinolytic system [14], we infected mice in which the activity of the fibrinolytic system was either impaired, i.e. C57BL/6 tPA deficient mice, or enhanced, i.e. C57BL/6 PAI-1 knock-out mice. First we demonstrated that *B. burgdorferi* infection did not influence fibrinolytic activity in citrate plasma in either mouse strain, or WT controls, as measured by amidolytic plasminogen activator activity assays (**Table 1**). Next, we showed that, compared to C57BL/6 WT mice, both C57BL/6 tPA and as PAI-1 knock-out mice display normal *Borrelia* numbers in various tissues two weeks (**Table 1**) and four weeks (data not shown) post infection, as detected by quantitative PCR and tissue culture (data not shown). In line with these data, phagocytotic capacity of C57BL/6 tPA and PAI-1 deficient neutrophils was comparable to that of WT mice (**Table 1**). Importantly, uPAR knock-out mice, regardless whether they were infected with *B. burgdorferi*, have comparable fibrinolytic activity to WT mice (data not shown). Together these data indicate that the impaired phagocytotic capacity of uPAR deficient mice, resulting in higher spirochete numbers upon *B. burgdorferi* infection in vivo, is not dependent on the role of uPAR in fibrinolysis.

**Table 1:** *B. burgdorferi* infection in WT, tPA −/− and PAI-1 −/− mice.

PA activity (in %)#	SHAM	*B. burgdorferi*
WT	91,2±1,4	89,6±0,8
tPA −/−	10,7±0,9^a^	9,9±2,1^c^
PAI-1 −/−	132,7±4,6^b^	141,7±3,9^d^
**Pathogen numbers** ## (*Borrelia FlaB* copies/1×10^6^ *β-actin* copies)		*ankle*
WT	-	1292±473
tPA −/−	-	636±366
PAI-1 −/−	-	560±149
		*skin*
WT	-	872±290
tPA −/−	-	724±301
PAI-1 −/−	-	1463±488
		*bladder*
WT	-	578±173
tPA −/−	-	967±310
PAI-1 −/−	-	367±125
**Phagocytosis index** $ (% pos * MFI)		
WT	ND	41015±5826
tPA −/−	ND	39828±3350
PAI-1 −/−	ND	49928±2752

**Note.** C57BL/6 WT, tPA and PAI-1 knock-out mice (6–8 per group) were inoculated with *B. burgdorferi* strain B31 or sham and sacrificed two weeks later.

# Plasminogen activator (PA) activity was measured in citrate plasma using amidolytic assays and expressed as a percentage.

## *B. burgdorferi* numbers were determined by quantitative PCR and expressed as described in **Figures 2** and **3**.

$ In addition, an in vitro phagocytosis assay was performed using naive mice (n = 6–8 per group) as described in **Figure 2**. Whole blood was incubated with viable CFSE-labeled *B. burgdorferi* for 60 minutes at 37 or 4°C as a control and phagocytosis was depicted as the phagocytosis index as described in **Figure 2**.

a,c PA activity was significantly lower in tPA knock-out mice compared to WT controls, regardless whether mice were inoculated with *B. burgdorferi* or sham, *p*<0,0001.

b,d PA activity was significantly higher in PAI-1 knock-out mice compared to WT controls, regardless whether mice were inoculated with *B. burgdorferi* or sham, *p*<0,0001.

Results represent the mean±SEM. Non-parametric statistical tests were used to analyze the differences between the groups. A *p*-value<0,05 was considered statistically significant.

### The effect of uPAR deficiency on the development of Lyme borreliosis

We assessed carditis severity in *B. burgdorferi* inoculated C57BL/6 uPAR knock-out and WT mice two and four weeks post infection. Two weeks post infection, in hematoxylin and eosin (H&E) stained sagittal sections of mouse hearts, we found comparable carditis severity scores in C57BL/6 WT and uPAR knock-out mice (**Figure S4A** and **B**). The localization and severity of carditis in our experiments using C57BL/6 mice appeared to be similar to the localization and carditis severities reported by ourselves and others using the same, relatively resistant, mouse strain [26]–[29]. Sham inoculated mice did not develop carditis (data not shown). We were unable to reliably score carditis four weeks post infection, since, as observed by others, at this stage, carditis was characterized by an organizing rather than ongoing inflammation (**Figure S4A**) [26]. However, in 4/8 uPAR deficient mice and 0/8 WT mice a mild active carditis, characterized by the presence of small cellular infiltrates at the aortic root, could still be observed 4 weeks post inoculation (Chi-square p = 0,021) (data not shown). By contrast, in 5/8 of WT mice and only in 2/8 uPAR deficient mice we observed organized inflammatory infiltrates, characterized by sharply delineated foci (**Figure S4A**) of mononuclear leukocytes situated in the atrial wall (Chi-square p = 0,0721). Together these findings suggest a difference with respect to the kinetics of the organization of carditis in C57BL/6 uPAR knock-out and WT mice. In line with the observed normal *Borrelia* numbers, in uPA, tPA and PAI-1 knock-out mice severity of carditis was comparable to that in WT mice (**Figure S4C** and **D**). Together these data demonstrate that, despite higher *B. burgdorferi* numbers, C57BL/6 uPAR knock-out mice develop carditis with a similar severity, albeit for a prolonged period of time, compared to WT controls. Finally, although we observed ankle swelling in both WT and uPAR C57BL/6 knock-out mice during the course of infection, histological examination of H&E stained section of tibiotarsi did not reveal any signs of arthritis 2, 4 or 6 weeks post infection (data not shown).

### The course of *B. burgdorferi* infection in uPAR deficient mice on a *B. burgdorferi* susceptible genetic background

**Figure 5:**
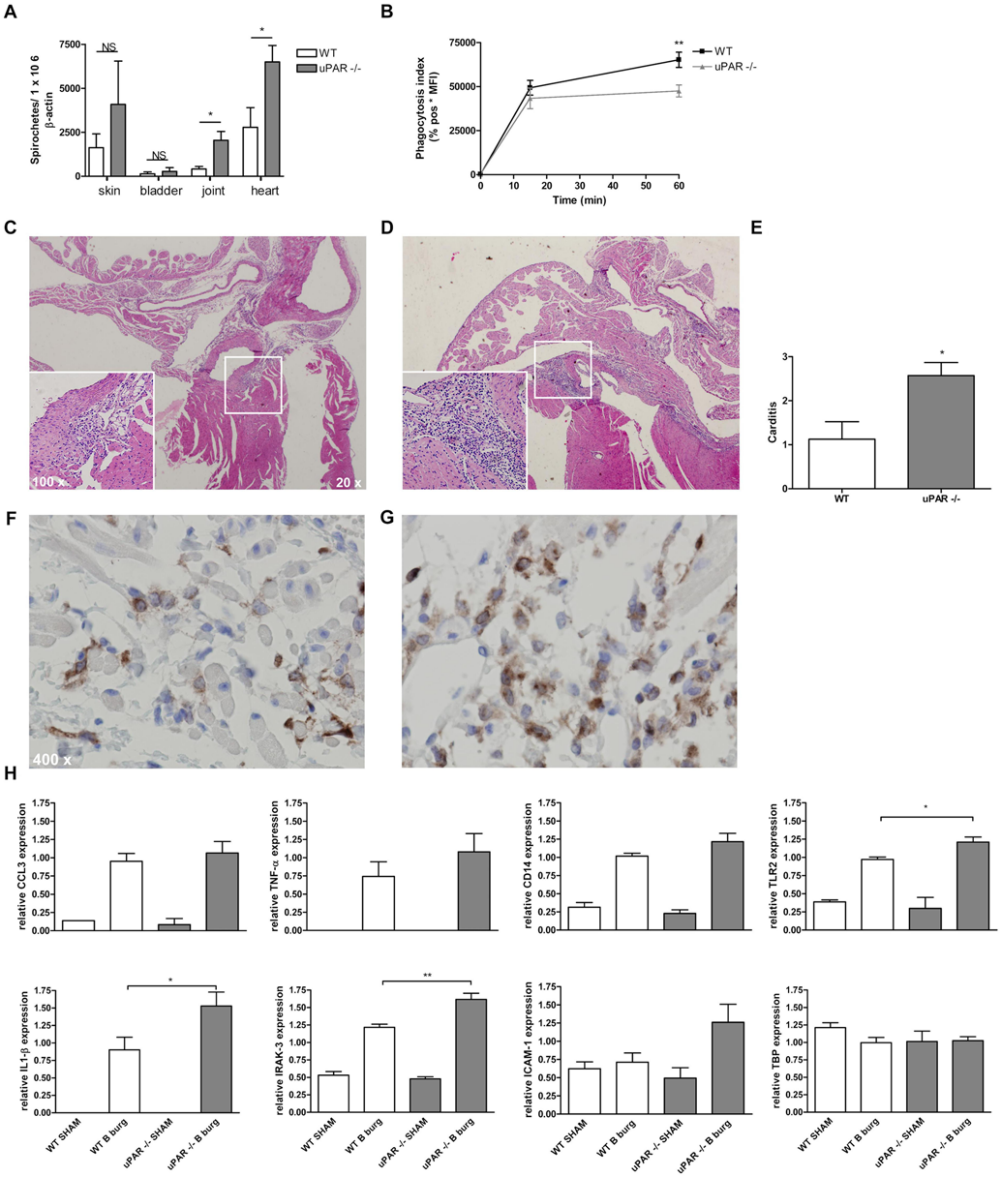
The course of Lyme borreliosis in uPAR knock-out mice on a *B. burgdorferi* susceptible mixed C57BL/6×C3H/HeN genetic background. (A) Urokinase receptor deficient mice on the mixed genetic background also display higher *B. burgdorferi* numbers compared to WT littermate controls. C57BL/6 mice were backcrossed twice to a C3H/HeN background. We intercrossed F2 mice and used the homozygous and nullizygous offspring (F2 homozygous uPAR deficient C57BL/6×C3H/HeN mice and WT littermate controls) for our experiments. Mice were inoculated with *B. burgdorferi* or sham and sacrificed two weeks post infection, DNA was extracted from the indicated tissues and samples were subjected to quantitative *Borrelia flab* and mouse *β-actin* PCR. *B. burgdorferi* numbers are depicted as described in Figure 2. Six to eight mice per group were used. (B) Urokinase receptor deficient leukocytes from mice on the mixed genetic background are not as capable of phagocytosing *B. burgdorferi* as are granulocytes from WT littermate controls. Phagocytosis assays with whole blood were performed as described in Figure 2. Six to eight mice per group were used. (C, D and E) Peak carditis in these uPAR −/− mice (D) is more severe compared to carditis in WT littermate controls (C). Mice were inoculated with *B. burgdorferi* and sacrificed two weeks post infection. Pictures of hematoxylin and eosin stained sagittal sections depict representative sections. Carditis was scored as described in Figure 4 within the same session (E). Six to eight mice per group were used. (F and G) The main cell involved in murine Lyme carditis is the macrophage. Representative pictures of F4/80 stained sagittal sections of hearts from *B. burgdorferi* infected uPAR deficient mice (G) and WT littermate controls (F). (H) More severe inflammation in *B. burgdorferi* infected uPAR deficient animals (n = 7) compared to WT littermate controls (n = 7) as measured by multiplex ligation-dependent probe amplification (MPLA). MLPA was performed on RNA obtained from half of sagittally dissected hearts from *B. burgdorferi* or sham inoculated mice. Depicted are mRNA expression of *TNF-α*, *CCL3*, *TLR2*, *CD14*, *IL1-β*, *IRAK3*, *ICAM1* and *TBP* (*housekeeping gene* [66]). Other genes included in the assay were *IL6*, *IL10*, *INF-γ*, *TFPI*, *F3*, *PROCR*, *SERPINE1P*, *PLAT*, *PLAUR*, *TLR4*, *TLR9 LY96*, *IRAK1*, *F2R*, *NFKB1a*, *NOS3*, *ITGA5*,*B2M*, *ITGAV*, *ITGAB3*, *TFRC*, *HIF1A*, *MMP2* and *HP*. Bars represent the mean±SEM. A *p*-value<0.05 was considered statistically significant. * indicating *p*<0,05; ** *p*<0,01.

To further investigate the effect of uPAR deficiency on the development of Lyme borreliosis symptoms we generated uPAR deficient mice on a more *Borrelia* susceptible genetic background. It is well-known that C57BL/6 mice are relatively resistant to *B. burgdorferi* and develop less severe symptoms after infection with the spirochete, and that C3H/HeN mice are more susceptible and develop more severe symptoms after infection with *B. burgdorferi* [30]. In addition, it has been described that F1 of WT C57BL/6 crossed with (x) C3H/HeN mice are intermediately sensitive to *B. burgdorferi* infection [30]. Therefore we investigated the course of Lyme borreliosis in F2 of C57BL/6×C3H/HeN uPAR knock-out mice and WT littermate controls. We first showed that, similar to uPAR knock-out mice on a pure C57BL/6 background, these mice harbor higher *Borrelia* numbers in multiple tissues compared to WT littermate controls two weeks post infection (**Figure 5A**), indicating that the lack of uPAR in these mice also resulted in impaired phagocytosis and increased pathogen burden. Indeed, in in vitro phagocytosis assays, compared to WT littermate controls, C57BL/6×C3H/HeN uPAR deficient neutrophils were significantly less capable of phagocytosing *B. burgdorferi* (**Figure 5B**). Strikingly, compared to WT littermate controls (**Figure 5C**), C57BL/6×C3H/HeN uPAR knock-out mice developed significantly more severe carditis (**Figure 5D**), reflected by influx of greater numbers of leukocytes in more and larger parts of cardiac tissue two weeks post infection (**Figure 5E**). As has been shown by others the main cells involved in inflammation were macrophages, as determined by F4/80 immunostaining (**Figure 5F** and **G**). By multiplex ligation-dependent probe amplification (MLPA), we detected significantly increased levels of interleukin (IL)-1β, IL-1 Receptor Associated Kinase (IRAK)-3, and toll-like receptor (TLR)2 mRNA in hearts from uPAR knock-out mice compared to WT littermate controls two weeks post infection (**Figure 5H**), consistent with the observed higher *B. burgdorferi* numbers and more severe cardiac inflammation in uPAR knock-out mice. Since, uPAR has also been shown to enhance migration of leukocytes towards the site of infection for some, but not all bacteria, in these mice we performed in vitro migration assays with WT and uPAR deficient macrophages (**Figure S5A**). We observed impaired migration of uPAR deficient macrophages to C5a (**Figure S5B**), however not to supernatant from a cardiomyoblastic rodent cell line (**Figure S5C**), compared to migration of WT macrophages, which is in line with our in vivo observations in C57BL/6 mice, In this in vitro setting, whether or not this cell line was stimulated with viable *B. burgdorferi* did not affect migration of WT and uPAR deficient macrophages, which might be due to production of both stimulating and inhibitory chemotactic stimuli of these cardiomyoblastic cells upon exposure to *B. burgdorferi*, as has been recently shown for neutrophils [31]. WT and uPAR deficient C57BL/6×C3H/HeN mice developed comparable ankle swelling during the course of *B. burgdorferi* infection (**Figure S5D**), however despite the more susceptible phenotype of these mice compared to C57BL/6 mice, these mice did not develop any histological signs of arthritis, as determined by hematoxylin and eosin staining, but also Ly6G - a marker for granulocytes - immunostaining (data not shown). In line with these data, post mortem radiological examination of the hind limbs did not reveal any signs of arthritis (**Figure S5E**).

## Discussion

Since its discovery approximately 30 years ago Lyme borreliosis has become the most important vector-borne disease in the Western world. We here demonstrate, to our knowledge for the first time, that uPAR plays an important role in the antibacterial innate immune response against *B. burgdorferi*. We show that uPAR expression is upregulated in response to *B. burgdorferi* on human and murine leukocytes both in vitro, as well in vivo. Importantly, we describe the role of uPAR in the immune response against *B. burgdorferi*. By using C57BL/6 WT and uPAR knock-out mice we show that uPAR plays an important role in phagocytosis of *B. burgdorferi* - a prerequisite for the eradication of the spirochete - by leukocytes. Moreover, experiments with C57BL/6 uPA, tPA and PAI-1 knock-out mice show that the mechanism by which uPAR is involved in the phagocytosis of *B. burgdorferi* is independent of ligation to uPA or uPAR's role in fibrinolysis. Finally, we show that, in mice relatively susceptible to *Borrelia* infection - mice on a mixed C57BL/6 and C3H/HeN background - uPAR deficiency also impaired phagocytotic capacity in vitro, which was associated with higher *B. burgdorferi* numbers, more local inflammation and more severe carditis, compared to WT littermate control animals, further underscoring the in vivo relevance of our findings. Together these data demonstrate an important role for uPAR in the innate immune response against, and the clearance of, the causative agent of Lyme borreliosis.

Earlier studies documented that membrane bound uPAR and uPAR mRNA are upregulated in human peripheral blood-derived monocytes and the human monocyte-like cell line U937 upon exposure to viable and heat-killed *B. burgdorferi* [12],[13]. We here show that viable *B. burgdorferi* induces upregulation uPAR (**Figure 1** and **Figure S1**), not only on murine and human monocytes, but also on macrophages and granulocytes in vitro. Notably, uPAR expression in response to *B. burgdorferi* in vivo has never been investigated. We here show that in skin from Lyme borreliosis patients with erythema migrans uPAR mRNA expression is significantly increased and could be readily detected by quantitative RT-PCR (**Figure 1**). Increased levels of uPAR are likely to be caused by influx of leukocytes to the site of the tick-bite. Indeed, in preliminary experiments in which we inoculated human skin ex vivo with viable *B. burgdorferi* - a model in which there is no influx of leukocytes [32] - we did not observe an increase in uPAR expression as determined by uPAR immunostaining on snap frozen sagittal skin sections (data not shown). Erythema migrans lesions are characterized by perivascular infiltrates in the dermis composed primarily of lymphocytes and macrophages [33]. We do not know which infiltrating cell type is responsible for the elevated uPAR levels, but based on our in vitro data we speculate that the macrophage is the most likely candidate. Indeed, macrophages from intraperitoneally *B. burgdorferi*-inoculated WT C57BL/6 mice did upregulate uPAR expression, further indicating that *Borrelia*-phagocyte interaction in vivo results in induction of uPAR expression (**Figure S1**). Upregulation of uPAR appeared not to be specific for *B. burgdorferi* since, in our in vitro experiments, other bacteria, i.e. *Klebsiella pneumoniae* and *Burkholderia pseudomallei*, also induce upregulation of uPAR to a similar extent (data not shown).

To investigate the role of uPAR in the immune response against *B. burgdorferi* and the course of murine Lyme borreliosis we inoculated C57BL/6 WT and uPAR knock-out mice with *B. burgdorferi*. We demonstrate by quantitative PCR and culture that mice lacking uPAR display significantly increased *B. burgdorferi* numbers in all tissue examined, indicative of a more disseminated infection (**Figure 2**), although also in these mice there appeared to be clearance of *B. burgdorferi*, as suggested by lower numbers 4 weeks compared to 2 weeks post infection. The increased *B. burgdorferi* burden in uPAR deficient mice was underscored by a more abundant, putatively reactive, IgG response (**Figure 2**). The role of uPAR in leukocyte adhesion and migration, leading to recruitment of these cells to the site of infection, has been the topic of investigations for many years. Several in vivo studies show that migration of uPAR deficient leukocytes is impaired in response to, for example, *Pseudomonas aeruginosum* [22] and *Streptococcus pneumoniae* [23]. In other studies, e.g. in *E. coli*-induced peritonitis [20] and pyelonephritis [21] uPAR deficiency did not affect leukocyte recruitment, indicating that the role of uPAR in migration of leukocytes is dependent on the pathogen, the site of infection and the disease model. Interestingly, in the mouse model for Lyme borreliosis uPAR is not crucially involved in migration of leukocytes to *B. burgdorferi* infected tissues, as indicated in our in vivo migration experiments (**Figure 3**). Strikingly, the fact that we observed more macrophages 32 hours after injection with *B. burgdorferi* in uPAR knock-out skin compared to WT controls, but no differences in H&E staining, suggests that the quality of the inflammatory infiltrate is affected rather than the quantity; presumably due to higher *B. burgdorferi* numbers in the uPAR knock-out mice. Interestingly, recently it was shown that uPAR also facilitates phagocytosis of the gram-negative bacterium *E. coli* by neutrophils [19],[21]. We here show, by fluorescent microscopic assays, and FACS-based phagocytosis assays, that both uPAR deficient granulocytes and macrophages are significantly less capable of phagocytosing viable spirochetes (**Figure 2** and **Figure S2** and **S3**). Importantly, uPAR deficiency did not affect binding of the spirochete to the surface of leukocytes (**Figure 2**). In addition, in an in vitro killing assay uPAR appeared not to be involved in killing of the spirochete following phagocytosis (data not shown), indicating that uPAR is involved strictly in the process of internalization of *B. burgdorferi* by leukocytes. Others have previously shown that phagocytosis of spirochetes by immune cells can be crucial for adequate cytokine induction and leukocyte activation [34]–[36]. We did not observe defects in pro-inflammatory cytokine production in uPAR deficient leukocytes when stimulated in vitro with *B. burgdorferi*. In contrast to the studies described above our results describe more subtle differences in phagocytotic capacity between WT and uPAR deficient leukocytes; we demonstrate diminished, but not absent, phagocytosis in uPAR deficient macrophages compared to WT controls.

The role of uPAR in phagocytosis of *B. burgdorferi* appeared to be independent of uPA and uPAR's role in the fibrinolytic system, since in our phagocytosis assays uPA, tPA and PAI-1 knock-out mice all displayed normal phagocytotic capacity of the spirochete compared to WT mice (**Figure 3** and **Table 1**). In addition, in vivo experiments clearly show that when these mice were inoculated with *B. burgdorferi* and sacrificed two weeks post infection, normal *B. burgdorferi* numbers were detected (**Figure 3** and **Table 1**). There are numerous in vitro studies reporting that *B. burgdorferi* interacts with the fibrinolytic system (reviewed in [6]). Extrapolating these data to the in vivo situation, this interaction, mainly through binding to host derived plasminogen, was thought to enable the spirochete to penetrate tissues, the blood-brain barrier and migrate through the extracellular matrix [8], [37]–[39]. Indeed, for the spirochetal causative agent of relapsing fever, using plasminogen knock-out mice, it has been clearly shown that plasminogen is required for dissemination of the spirochete to the heart and brain in vivo [40]. To our knowledge, for *B. burgdorferi* however, there is only one previously published study that describes the effect of diminished fibrinolytic activity on the course of *B. burgdorferi* infection in vivo [7]. In this study, in which plasminogen deficient mice were used, plasminogen was shown to be important for dissemination of the spirochete within the feeding tick. Strikingly, despite a short-lived spirochetemia, there were no differences in *B. burgdorferi* numbers in any of the tissues examined in plasminogen knock-out compared to WT mice at several time points post infection [7]. In line with these data, our results demonstrate that the fibrinolytic system per se does not affect the course of *B. burgdorferi* infection. Strikingly, we here show that one of the key players in the fibrinolytic system, uPAR, independently of ligation to uPA or its presumptive role in fibrinolysis, is importantly involved in the course of experimental murine Lyme borreliosis.

The fact that we show that the requirement of uPAR in phagocytosis of *B. burgdorferi* is independent of uPA or uPAR's role in the fibrinolytic system suggests that the requirement of uPAR in internalization of *Borrelia* is dependent on interaction of uPAR with other cell surface molecules. Indeed, uPAR has been shown to facilitate various leukocyte functions, among which adhesion, migration and phagocytosis through interaction with αβ-integrins and other cell surface molecules, but also vitronectin [14],[15]. This implies a role for uPAR as a signaling receptor. However, because uPAR is a glycosyl-phosphatidylinositol linked receptor and lacks a cytosolic domain it needs to form functional transmembrane units with other molecules, such as multiple αβ-integrins, G-protein-coupled receptors, and caveolin in order to induce intracellular signaling events leading to cytoskeleton rearrangements and consequent cell movement [14],[15]. Since both uPAR and *B. burgdorferi* share many molecules with which they can interact, for example αβ-integrins and vitronectin, it will be challenging to identify the surface molecule with which uPAR associates to facilitate phagocytosis of *B. burgdorferi*.

When we infected uPAR knock-out mice on a mixed C57BL/6 and C3H/HeN background with *B. burgdorferi* these mice exhibited higher *B. burgdorferi* numbers in cardiac tissue two weeks post infection compared to WT littermate controls, which was also associated with decreased phagocytosis of *B. burgdorferi*. Strikingly, in these mice we observed a significantly increased influx of leukocytes, predominantly macrophages, at the atrioventricular junction and at the aortic root compared to WT littermate controls (Figure 5), further indicating that uPAR is not required for migration of leukocytes in response to *B. burgdorferi*, which was also underscored by the in vitro migration assays (**Figure S5**). Furthermore, our data indicate that, although the underlying mechanisms appeared to be the same, the consequences of uPAR deficiency for the course of murine Lyme borreliosis are dependent on the genetic background of the host. Others have shown that C57BL/6 and C3H/HeN mice harbor similar *B. burgdorferi* numbers after infection, but the severity of symptoms was more pronounced in C3H/HeN mice [30], indicating that the extent of the immune response that is mounted against the spirochete is dependent on the genetic background of the host. Indeed, we have demonstrated that uPAR deficiency in *Borrelia* resistant C57BL/6 mice leads to higher *B. burgdorferi* loads, but to comparable, albeit longer-lived active carditis compared to WT controls. By contrast, uPAR deficient mice on a more susceptible mixed C57BL/6×C3H/HeN background also exhibited higher *B. burgdorferi* numbers, but more pronounced influx of leukocytes and more severe carditis. Local cytokines and chemokines induced by *B. burgdorferi* are thought to mediate Lyme carditis. A cytokine that has been implicated to be of paramount importance for local inflammation and migration of leukocytes is IL-1β [41]–[43]. Also in *B. burgdorferi* infected mice and patients IL-1β has been shown to be upregulated in heart or joints [44]–[47] Interestingly, by MLPA we found significantly higher levels of mRNA coding for IL-1β, IL-1 receptor associated kinase (IRAK)-3 (predominantly expressed in macrophages) and TLR2 (the TLR preferentially recognizing *B. burgdorferi* lipoproteins) in hearts from *B. burgdorferi* infected uPAR knock-out mice on the mixed genetic background compared to WT littermate controls (**Figure 5**). Interestingly, in previous studies we showed that (human) peripheral blood-derived dendritic cells stimulated with the TLR2 ligand lipoteichoic acid (LTA) or viable *B. burgdorferi* produced high levels of IL-1β [48]. One could argue against the use of F2 mice in our studies, however the fact that F1 WT C57BL/6×WT C3H/HeN mice are already intermediate susceptible to *Borrelia* infection [30], encouraged us to perform our experiments with F2 mice.

Together, our data suggest that decreased phagocytosis of *B. burgdorferi* by uPAR deficient leukocytes (**Figure 2**, **Figure S2** and **Figure 5**) resulted in higher local and systemic *B. burgdorferi* numbers, apparent in later stages post inoculation (**Figure 2** and **5**). Early in infection increased *Borrelia* numbers might enhance local skin innate immune responses in uPAR knock-out mice resulting in significantly more influx of phagocytes, as shown by in vivo and in vitro migration assays (**Figure 3** and **Figure S5**). The increased influx of phagocytes might temporarily compensate for the impaired phagocytosis in uPAR deficient leukocytes and temporarily control dissemination of *Borrelia*. However, as shown by our in vivo data, in uPAR knock-out mice, *B. burgdorferi* will eventually manage to disseminate resulting in increased *B. burgdorferi* numbers in distant organs during later stages of infection compared to WT animals (**Figure 2** and **5**). In uPAR knock-out mice on the more susceptible genetic background, these higher *Borrelia* numbers were associated with an increased influx of leukocytes, as demonstrated by pathology of mouse hearts (Figure 5). Since, uPAR deficient leukocytes are as capable as WT leukocytes in producing pro-inflammatory cytokines upon exposure to *B. burgdorferi* (Figure 2), this could explain the increased inflammation and tissue damage observed in these mice compared to WT controls (Figure 5). Therefore, we postulate that in WT mice, upon *B. burgdorferi* infection, leukocytes upregulate uPAR (Figure 1 and Figure S1), which facilitates phagocytosis of the spirochete, reducing the number of disseminating spirochetes and thereby limiting the extent and severity of inflammation of distant sites, such as the heart. In conclusion, we here show that uPAR is importantly involved in the host defense against *B. burgdorferi* in vivo by a mechanism that is independent of binding of uPAR to uPA or its role in the fibrinolytic system.

## Materials and Methods

### Mice, spirochetes and infection

Specific pathogen-free wildtype C57BL/6 mice were purchased from Harlan Sprague Dawley Inc. (Horst, The Netherlands) and uPAR knock-out C57BL/6 mice were purchased from Jackson Laboratories (Bar Harbor, ME) [49]. In addition C57BL/6 uPAR knock-out mice were backcrossed twice to a C3H/HeN - purchased from Jackson Laboratories – background, generating F2 C57BL/6×C3H/HeN heterozygous uPAR deficient mice. F2 mice were crossed among each other to generate homozygous C3H/HeN×C57BL/6 uPAR knock-out mice and WT littermate controls. uPA, tPA and PAI-1 knock-out mice were also purchased from Jackson Laboratories. All mice were bred in the animal facility of the Academic Medical Center (Amsterdam, The Netherlands). Age-and sex-matched animals were used in each experiment and the Animal Care and Use Committee of the University of Amsterdam approved all experiments. Six to eight-week old mice were infected by intradermal syringe inoculation with 1×10^6^ *B. burgdorferi* sensu stricto strain B31 clone 5A11 [50], that had previously been recovered from an experimentally infected mouse [29]. Spirochetes were cultured in BSK-II medium, enumerated and inoculated in the midline of the back or with BSK-II medium as a control (SHAM), as described previously [29],[51]. Mice were sacrificed by bleeding from the inferior vena cava at the indicated time points, i.e. 2, 4 (or 6 weeks) post infection. Heparin or citrate plasma was stored at −20°C for future use. Skin (inoculation site), urinary bladder, heart and tibiotarsi were saved for histopathological examination, culture or quantitative Polymerase Chain Reaction (q-PCR).

### Q-PCR

DNA from murine tissues was obtained with the DNeasy KIT (Qiagen, Venlo, The Netherlands) as previously described [29]. Quantitative PCR detecting *Borrelia flaB* and mouse *β-actin* was performed, as described previously [29]. Standards consisted of dilutions of genomic DNA from *B. burgdorferi* or mouse *ß-actin* (252 bp) cloned into the PCR2.1-TOPO vector (Invitrogen, Breda, The Netherlands), as described previously [29],[51]

### Arthritis, paw swelling and radiological examination

Histopathological changes in tibiotarsi were assessed as previously described [29],[52]. We monitored ankle swelling of both tibiotarsal joints using a Mitutoyo pressure controlled microcaliper (Mitutoyo, Kanagawa, Japan). Measurements were performed several times throughout the course of the infection by the same observer blinded to the experimental design. Lastly, we performed post mortem radiological examination of formalin fixed right hind paws, as described previously [53].

### Carditis

Five µm-thick paraffin embedded sections of sagittally dissected hearts were processed and H&E stained by routine histological techniques. Carditis was scored on a scale from 0 to 3 by a pathologist blinded to the experimental design, essentially as previously described [27],[28],[51], with 0: no carditis; 1: mild carditis; 2: moderate carditis and 3: severe carditis. As described previously [26], 2 weeks post infection, carditis was characterized by disperse inflammation at the atrioventricular junction and aortic root, where as four weeks post infection, organizing inflammation was characterized by the presence of sharply delineated foci of >50 mononuclear cells in the atrial walls. An F4/80 immunostaining (BMA Biomedicals, Augst, Switzerland) was performed to detect influx of macrophages [54].

### Multiplex ligation-dependent probe amplification

MLPA was performed in essence as described before [55]. The genes that were analyzed are listed in the figure legend for **Figure S3**. Equal amounts of mRNA were included per reaction and all samples were tested in a single experiment using the same batch of reagents. The levels of mRNA for each gene were expressed as a normalized ratio of the peak area of the fluorescent intensity (in arbitrary units) and divided by the cumulative peak area of all genes in the assay, resulting in the relative abundances of mRNAs of the genes of interest [56].

### Whole cell *B. burgdorferi* ELISA

*Borrelia burgdorferi* sensu stricto strain B31 specific total immunoglobulin (Ig)G and IgG subclasses were measured in heparin plasma from infected animals and controls by ELISA as described previously [29]. All measurements were performed in duplicate.

### Amidolytic assays of PA activity

Plasminogen activator (PA) activity was measured as a measure for the activity of the fibrinolytic system using an amidolytic assay as described earlier [23],[57]. Briefly, citrate plasma was incubated with S-2251 (Chromogenix, Mölndal, Sweden), plasminogen and cyanogen bromide fragments of fibrinogen (Chromogenix, Milano, Italy). Conversion of plasminogen to plasmin was assessed by subsequent conversion of the chromogenic substrate S-2251 and was detected with a spectrophotometer.

### Stimulation assays

Whole blood and peritoneal macrophages from three naive uPAR knock-out or WT mice were harvested as described [58]. Briefly, 1×10^5^ adherent macrophages and heparinized whole blood were stimulated in duplo in 96-well microtiter plates (Greiner) with 1×10^6^ or 1×10^7^ viable *B. burgdorferi* suspended in Roswell Park Memorial Institute (RPMI) 1640 medium or medium as a negative control for 16 h. Supernatants were collected and stored at −20°C until cytokine production was measured by CBA. For assessment of uPAR expression by fluorescence activated cell sorter (FACS), cells were harvested and stained with murine anti-CD87-Phycoerythrin (PE) (BD Pharmingen, Maarssen, The Netherlands). To assess uPAR expression on specific cells, cells were double-stained with anti-GR1-fluorescein isothiocyanate (FITC) (BD Pharmingen) (granulocytes) or F4/80-allophycocyanin (APC) (BD Pharmingen) (monocytes and macrophages). In addition, in non-phagocytosing cells, i.e. CD4^+^ and CD8^+^ T cells - stained with anti-CD3-APC (BD Pharmingen) and anti-CD4-FITC or anti-CD8-PerCP respectively (BD Pharmingen) - we also assessed uPAR expression by FACS analysis. Similarly, uPAR expression on human cells derived from heparinized whole blood was analyzed with a human biotin-labeled antibody against uPAR (R&D Systems, Minneapolis, MN) in combination with streptavidin conjugated to PE; cells were triple-stained with also anti-CD15-APC (BD Pharmingen) (granulocytes) and anti-CD14-Cy-Chrome 5 (Cy5) (BD Pharmigen) (monocytes) (BD Pharmingen). Human macrophages were generated as described previously [59]. Briefly, human peripheral blood derived mononuclear cells were isolated from buffy coats by centrifugation over a Ficoll-Paque gradient. Subsequently, adherent monocytes were cultured in X-VIVO medium (BioWhittaker, Walkersville, MD) with 1% heat-inactivated autologous plasma to allow for differentiation to human monocyte-derived macrophages in 7 days. Antibodies were used in concentrations recommended by the manufacturer and FACS analysis was performed using the BD FACScalibur (BD Biosciences, Breda, The Netherlands). Endotoxin concentration in the *B. burgdorferi* culture media was approximately 1 IU/ml, as determined by a Cambrex QCL LAL assay (Cambrex). We established that the maximal amount of LPS that could have possibly contaminated the final *Borrelia* preparation used for the in vitro stimulations - after extensive washing and resuspension in different cell culture media - was insufficient to influence uPAR expression (data not shown). In a separate experiment viable *B. burgdorferi* (1×10^8^) were injected into the peritoneal cavity of C57BL/6 WT or uPAR knock-out mice for one hour. Hereafter cells were harvested, stained for F4/80, and CD87 (uPAR) expression was measured by FACS analysis.

### Detection of uPAR mRNA expression in human samples

Transcutaneous skin biopsies were collected from healthy volunteers, i.e. non-inflammed skin, or patients with active Lyme erythema migrans at the Academic Medical Center, Amsterdam, The Netherlands and New York Medical College, NY. IRB approval was obtained from both institutes. All Lyme patient skin samples were tested positive for *B. burgdorferi* spirochetes by in vitro culture and PCR. Skin samples were frozen-ground to fine powder using a china grinder and RNA was extracted using the TRIZOL reagent from Invitrogen (Carlsbad, CA, U.S.A). RNA samples were treated with TURBO DNase (Applied Biosystems, Foster City, CA, U.S.A) to remove DNA contaminants. RNA was then converted to cDNA using an Affinity Script kit (Stratagene, La Jolla, CA, U.S.A). Quantification of uPAR was performed by Taqman PCR (Applied Biosystems) and normalized to β-actin (*ACTB*). The primers and probes used for uPAR were forward 5′AATCCTGGAGCTTTGAAAATCT 3′, reverse 5′CCACTTTTAGTACAGCAGGAGA 3′, and probe 5′6FAM-ACTGCCGAGGCCCCATGAATC 3′-TAMRA. Human β-actin primers and probe were inventoried products of Applied Biosystems.

### Phagocytosis assays

Phagocytosis assays were performed in essence as described before [60]–[62]. Viable *B. burgdorferi* were labeled with carboxyfluorescein diacetate succinimidyl ester (CFSE, Invitrogen) as described by others [63] or heat-inactivated (30 min at 56°C) non-motile, but intact, *B. burgdorferi* were labeled with fluorescein isothiocyanate (FITC). Adhered peritoneal macrophages (derived from 6–8 mice per group) were incubated with CFSE-labeled *B. burgdorferi* (Cell∶*Borrelia* = 1∶50) in serum-free RPMI 1640 medium in 24-well microtiter plates (Greiner, Alphen a/d Rijn, The Netherlands) for 0, 15 and 60 minutes at 37°C. Phagocytosis was stopped by transferring the cells to 4°C. Extracellular signal of *B. burgdorferi* was eliminated by addition of a quenching solution for one minute - containing Trypan blue that absorbs the fluorescence emission of both FITC and CFSE (Orpegen, Groningen, The Netherlands; [62].) - and three washes with ice-cold PBS. For each sample and each time point 4°C controls were performed, however there was hardly any phagocytosis detectable under these conditions (data not shown). Cells were resuspended in FACS buffer (PBS supplemented with 0,5% bovine serum albumin (BSA), 0,01% NaN3 and 0,35 mM EDTA) followed by FACS analysis. At 37°C the majority of spirochetes was internalized as was determined by control experiments in which we did not add the quenching solution (data not shown). Similarly, to determine neutrophil phagocytosis capacity, 50 µl of whole blood was incubated with 2×10^6^ viable CFSE-labeled *B. burgdorferi* for the indicated time, after which quenching solution was added for one minute and samples were washed twice with ice-cold FACS buffer. Thereafter cells were incubated with BD Lyse/Fix solution (BD Biosciences) and neutrophils were labeled using anti-Gr-1-PE (BD Pharmingen). Live cells were electronically gated and phagocytosis was determined using FACS. The phagocytosis index of each sample was calculated as previously, described: (mean fluorescence intensity (MFI)×percentage (%) positive cells) at 37°C minus (MFI×% positive cells) at 4°C [64],[65].

### Migration assays

In vitro migration experiments with murine peritoneal macrophages from WT and uPAR knock-out mice were performed essentially as described [64],[65]. Prior to experimentation cells were labeled with CellTracker Green (Molecular Probes, Eugene, Or) in serum-free Dulbecco's modified Eagle's medium (DMEM). The dye was fixed by 1 h incubation in DMEM plus 10% FCS. Thereafter cells were washed and resuspended in serum-free medium and transferred to 3 µM pore size HTS FluoroBlok Cell Culture Inserts (BD Falcon) which were inserted in fitting 24-well plates containing various attractants (*B. burgdorferi*, activated complement factor 5 (C5a)) also in DMEM serum-free medium. Fluorescence, representing the number of cells on the bottom side of the insert, was read every 2 min on a Series 4000 CytoFluor Multi-Well Plate Reader (Perseptive Biosystems, Framingham, MA). Raw fluorescence data were corrected for background fluorescence and no-attractants controls were subtracted at each measured time point to correct for random migration. Migration start points were set to zero. To mimic the in vivo situation more closely we also performed experiments with an embryonic rodent heart-derived cell line, H9c2 cells (CRL-1446, American Type Culture Collection, Queens Road, Teddington, UK). These cardiomyoblasts were maintained in DMEM with 10% foetal bovine serum (FBS). Prior to experimentation, cells were washed and resuspended in serum-free DMEM and incubated with viable *Borrelia* (Cell∶*Borrelia* = 1∶50) or medium as a control for 16 h. The supernatants were centrifuged for 5 minutes at 1200×*g* to remove cells and other particles, followed by centrifugation at 4000×*g* for 15 minute to remove the spirochetes. Supernatants were used undiluted or diluted (data not shown) as chemoattractants in the indicated experiments. All experiments were performed in duplo or in triplo and repeated three times. In addition, we also assessed migration of leukocytes in skin from C57BL/6 WT and uPAR deficient mice in response to *B. burgdorferi* in vivo (n = 5 per group). In these set of experiments we intradermally injected C57BL/6 WT mice with 1×10^6^ *B. burgdorferi* in PBS in the midline of the neck and mice were sacrificed 0, 6 or 32 hours post inoculation. Control animals were injected with PBS. Skin was harvested, formalin fixed and imbedded in paraffin. Five µm-thick sagittal skin sections were processed and H&E, Ly6G and F4/80 stained by routine histological techniques [54]. The control animals did no display influx of leukocytes (data not shown). Slides were scored for influx of leukocytes by an independent pathologist who was blinded to the experimental design. Influx was semi-quantitatively scored on a scale from 0–3, with 0 being no, 1 mild, 2 moderate, and 3 being severe diffuse infiltration.

### Statistical analysis

Differences between the groups were analyzed using the two-sided non-parametric Mann-Whitney U test (Graphpad Prism Software version 4.0, San Diego, CA). Where indicated a two-sided Chi-square indicated was applied. Data are presented as the mean±standard errors of the mean (SEM). A *p* value of<0.05 was considered significant, where * indicated *p*<0,05, ** *p*<0,01 and *** p<0,001. For ECG data statistical analysis was performed using a multivariate repeated measurements model (SPSS statistics software 17.0).

## Supporting Information

Figure S1 *Borrelia burgdorferi* induces upregulation of the urokinase receptor on leukocytes in vitro and in vivo. (A) Viable *B. burgdorferi* induces uPAR expression on ex vivo generated human macrophages. Cells were incubated with viable *B. burgdorferi* for 16 hours. Thereafter cells were stained with anti-CD87 (uPAR), electronically gated and analyzed by FACS analysis. Representative cytograms and histograms are shown. (B) Viable *B. burgdorferi* induces uPAR expression on murine granulocytes. Whole blood was incubated with viable *B. burgdorferi* for 16 hours. Erythrocytes were lysed, cells were co-stained with anti-GR-1 and anti-CD87 (uPAR), electronically gated and analyzed by FACS analysis. Representative cytograms and histograms are shown. (C) Viable *B. burgdorferi* (1×10^8^) were injected into the peritoneal cavity of C57BL/6 WT (n = 6) or uPAR knock-out (n = 4) mice for one hour. Hereafter cells were harvested, stained for F4/80, and CD87 (uPAR) expression was measured by FACS analysis. A *p*-value<0,05 was considered statistically significant. * indicating *p*<0,05; ** *p*<0,01. (D) In non-phagocytosing cells, i.e. CD4^+^ and CD8^+^ T cells - doublestained with anti-CD3-APC (BD Pharmingen) and anti-CD4-FITC and anti-CD8-PerCP, respectively - we also assessed CD87 (uPAR) expression upon stimulation with *B. burgdorferi* by FACS analysis. Error bars represent the mean of triplicates within one experiment±SEM.
(0.81 MB JPG)

Figure S2 Impaired phagocytosis of *B. burgdorferi* by uPAR deficient leukocytes. (A and B) Representative cytograms (A) and histograms from phagocytosis assays of *B. burgdorferi* by WT and uPAR deficient whole blood in time (B). Assays were performed as described in **Figure 2**. After the assays whole blood was lysed and stained with anti-GR-1 (granulocytes). Marker (M)1 encompasses positive cells.
(0.73 MB JPG)

Figure S3 Confocal microscopy of *B. burgdorferi* phagocytosis. (A and B) Confocal microscopy confirmed that *B. burgdorferi* in in vitro phagocytosis assays were localized intracellularly. Cells incubated with CFSE-labeled *B. burgdorferi* were subjected to confocal microscopy. Nuclei of cells were stained with DAPI. In Panel (A) we depicted the widest transversal section of a segmented nucleus of a granulocyte stained with DAPI and a CFSE-labeled *B. burgdorferi* spirochete. Superimposing the brightfield image confirms the bacterium is localized intracellularly. Panel (B) shows another granulocyte and *B. burgdorferi* from different view points (left panel) and a stack movie (right panel) further verifying that we are assessing internalized bacteria in the in vitro phagocytosis assays. Note: The Figure S3 Powerpoint file should be saved in the same folder as the AVI file in order view the figure correctly. In addition, open the Powerpoint file in slideshow format.
(0.80 MB ZIP)

Figure S4 Carditis in WT, uPAR, uPA, tPA and PAI-1 knock-out mice. (A and B) Peak carditis in C57BL/6 uPAR −/− is of similar severity compared to WT controls, although active carditis persists longer in uPAR −/− mice. WT and uPAR −/− mice were inoculated with *B. burgdorferi* and sacrificed two or four week post infection. Sagittal sections of formalin fixed and paraffin embedded hearts were H&E stained. The severity two weeks post infection was scored by a pathologist blinded to the experimental design on a scale of 0–3, with 0: no carditis; 1: mild carditis; 2: moderate carditis and 3: severe carditis. Sham inoculated mice did not develop carditis (data not shown). Pictures depict representative sections. (C and D) Peak carditis in C57BL/6 uPA, tPA and PAI-1 knock-out mice is comparable to peak carditis in WT C57BL/6 mice infected with *B. burgdorferi*. Carditis was scored as described above. Six to eight mice per group were used and bars represent the mean±SEM. A *p*-value<0,05 was considered statistically significant.
(1.19 MB JPG)

Figure S5 Migration and arthritis in WT and uPAR knock-out mice on a *B. burgdorferi* susceptible genetic background. (A, B and C) Urokinase receptor deficient macrophages from mice on the mixed genetic background can migrate to cardiogenic stimuli just as well as macrophages from WT littermate controls. Migration of CellTracker Green labeled WT or uPAR deficient macrophages towards several chemotactic stimuli was investigated in vitro (A). As chemotactic stimuli we used *B. burgdorferi* or activated complement factor 5 (C5a) (B) and supernatant from the cardiomyoblastic rodent cell line H9c2 stimulated with *B. burgdorferi* or control medium for 16 hours prior to experimentation (C). All conditions were tested in duplo, in serum free DMEM medium without the addition of antibiotics, and migration was corrected for the no-attractant control. Graphs represent the mean of three independent experiments±SEM. The fluorescent signal in the lower chamber (indicative of migration) was measured in real time every two minutes (cycli). (D and E) Only edema, no arthritis in *B. burgdorferi* infected uPAR knock-out mice (n = 7) and *B. burgdorferi* infected WT littermate controls (n = 8). Ankle swelling was measured using a microcaliper during the course of *B. burgdorferi* infection (D). In this particular experiment mice were monitored for three weeks. Post mortem, but before decalcification, radiological examination of the right hindlimb was performed (E). No differences between sham inoculated and *B. burgdorferi* infected animals were observed. A *p*-value<0,05 was considered statistically significant. * indicating *p*<0,05.
(0.47 MB JPG)
