# Supplementary figures and images for: The Urokinase Receptor (uPAR) Facilitates Clearance of Borrelia burgdorferi
Source: PLoS Pathog. 2009 May 22;5(5):e1000447. doi: 10.1371/journal.ppat.1000447 (PMC2678258; doi:10.1371/journal.ppat.1000447)

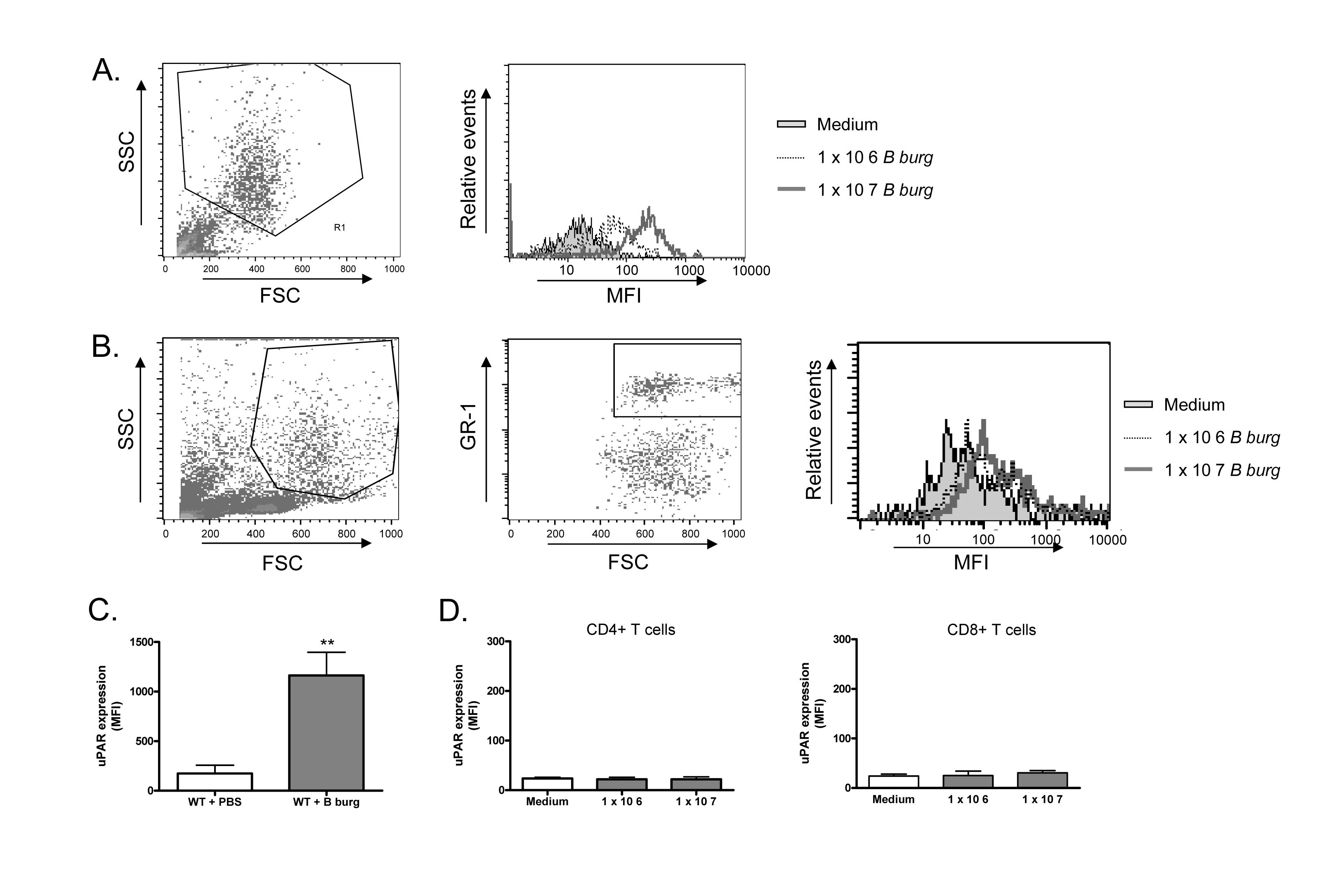

Supplement: Figure S1 — Borrelia burgdorferi induces upregulation of the urokinase receptor on leukocytes in vitro and in vivo. (A) Viable B. burgdorferi induces uPAR expression on ex vivo generated human macrophages. Cells were incubated with viable B. burgdorferi for 16 hours. Thereafter cells were stained with anti-CD87 (uPAR), electronically gated and analyzed by FACS analysis. Representative cytograms and histograms are shown. (B) Viable B. burgdorferi induces uPAR expression on murine granulocytes. Whole blood was incubated with viable B. burgdorferi for 16 hours. Erythrocytes were lysed, cells were co-stained with anti-GR-1 and anti-CD87 (uPAR), electronically gated and analyzed by FACS analysis. Representative cytograms and histograms are shown. (C) Viable B. burgdorferi (1×10^8^) were injected into the peritoneal cavity of C57BL/6 WT (n = 6) or uPAR knock-out (n = 4) mice for one hour. Hereafter cells were harvested, stained for F4/80, and CD87 (uPAR) expression was measured by FACS analysis. A p-value<0,05 was considered statistically significant. * indicating p<0,05; ** p<0,01. (D) In non-phagocytosing cells, i.e. CD4+ and CD8+ T cells - doublestained with anti-CD3-APC (BD Pharmingen) and anti-CD4-FITC and anti-CD8-PerCP, respectively - we also assessed CD87 (uPAR) expression upon stimulation with B. burgdorferi by FACS analysis. Error bars represent the mean of triplicates within one experiment±SEM. (0.81 MB JPG) [file ppat.1000447.s001.jpg]

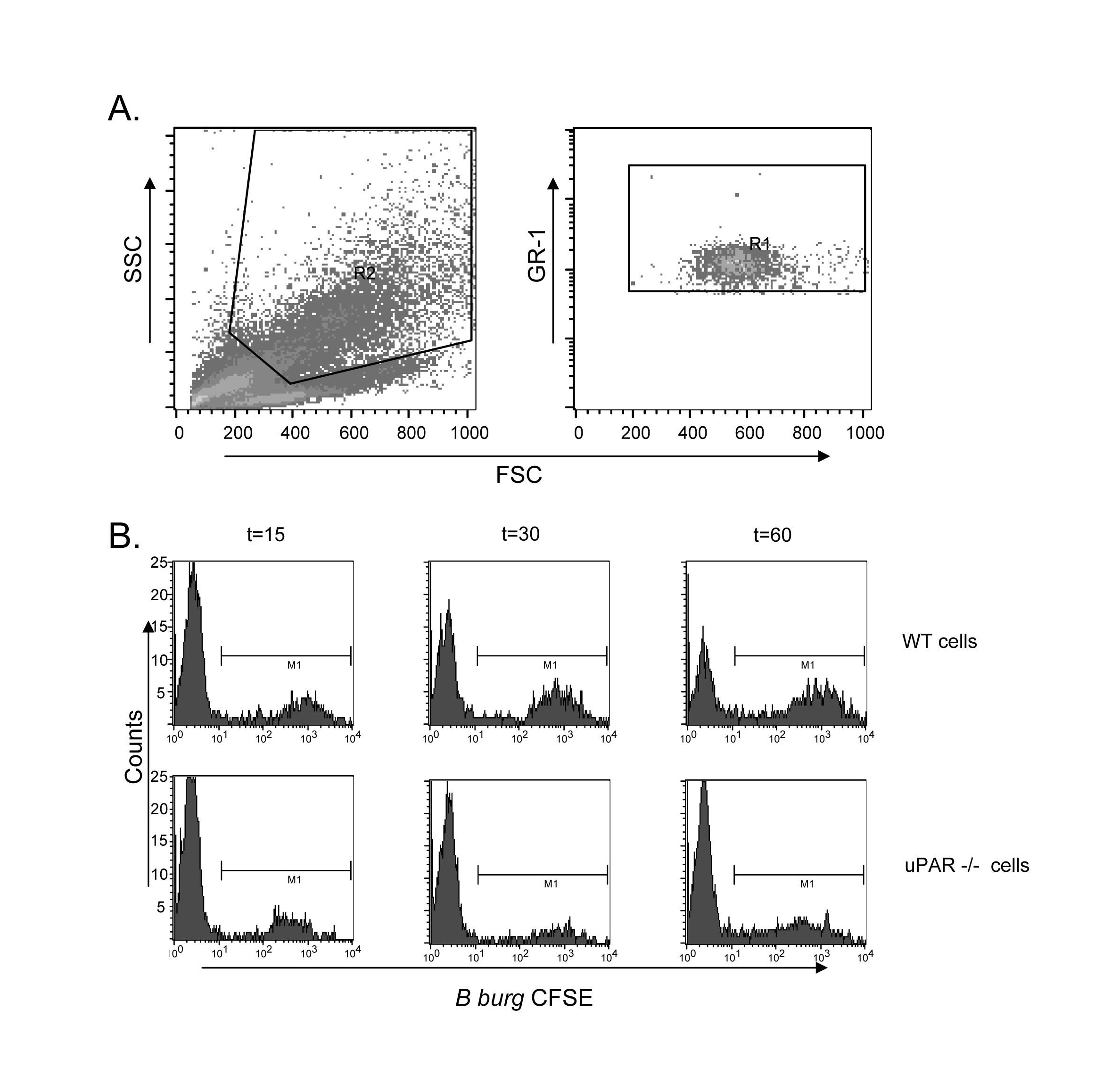

Supplement: Figure S2 — Impaired phagocytosis of B. burgdorferi by uPAR deficient leukocytes. (A and B) Representative cytograms (A) and histograms from phagocytosis assays of B. burgdorferi by WT and uPAR deficient whole blood in time (B). Assays were performed as described in Figure 2 . After the assays whole blood was lysed and stained with anti-GR-1 (granulocytes). Marker (M)1 encompasses positive cells. (0.73 MB JPG) [file ppat.1000447.s002.jpg]

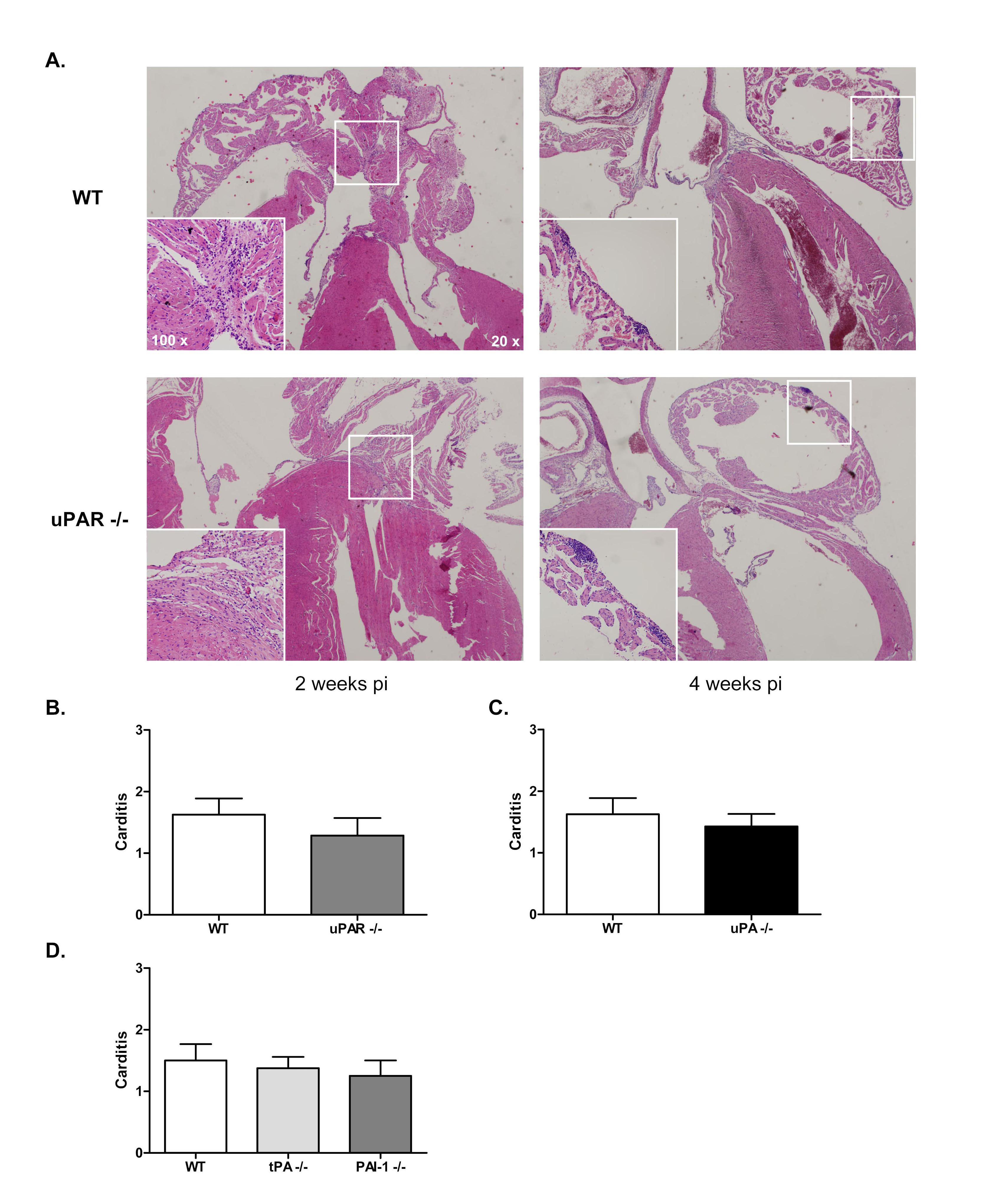

Supplement: Figure S4 — Carditis in WT, uPAR, uPA, tPA and PAI-1 knock-out mice. (A and B) Peak carditis in C57BL/6 uPAR −/− is of similar severity compared to WT controls, although active carditis persists longer in uPAR −/− mice. WT and uPAR −/− mice were inoculated with B. burgdorferi and sacrificed two or four week post infection. Sagittal sections of formalin fixed and paraffin embedded hearts were H&E stained. The severity two weeks post infection was scored by a pathologist blinded to the experimental design on a scale of 0–3, with 0: no carditis; 1: mild carditis; 2: moderate carditis and 3: severe carditis. Sham inoculated mice did not develop carditis (data not shown). Pictures depict representative sections. (C and D) Peak carditis in C57BL/6 uPA, tPA and PAI-1 knock-out mice is comparable to peak carditis in WT C57BL/6 mice infected with B. burgdorferi. Carditis was scored as described above. Six to eight mice per group were used and bars represent the mean±SEM. A p-value<0,05 was considered statistically significant. (1.19 MB JPG) [file ppat.1000447.s004.jpg]

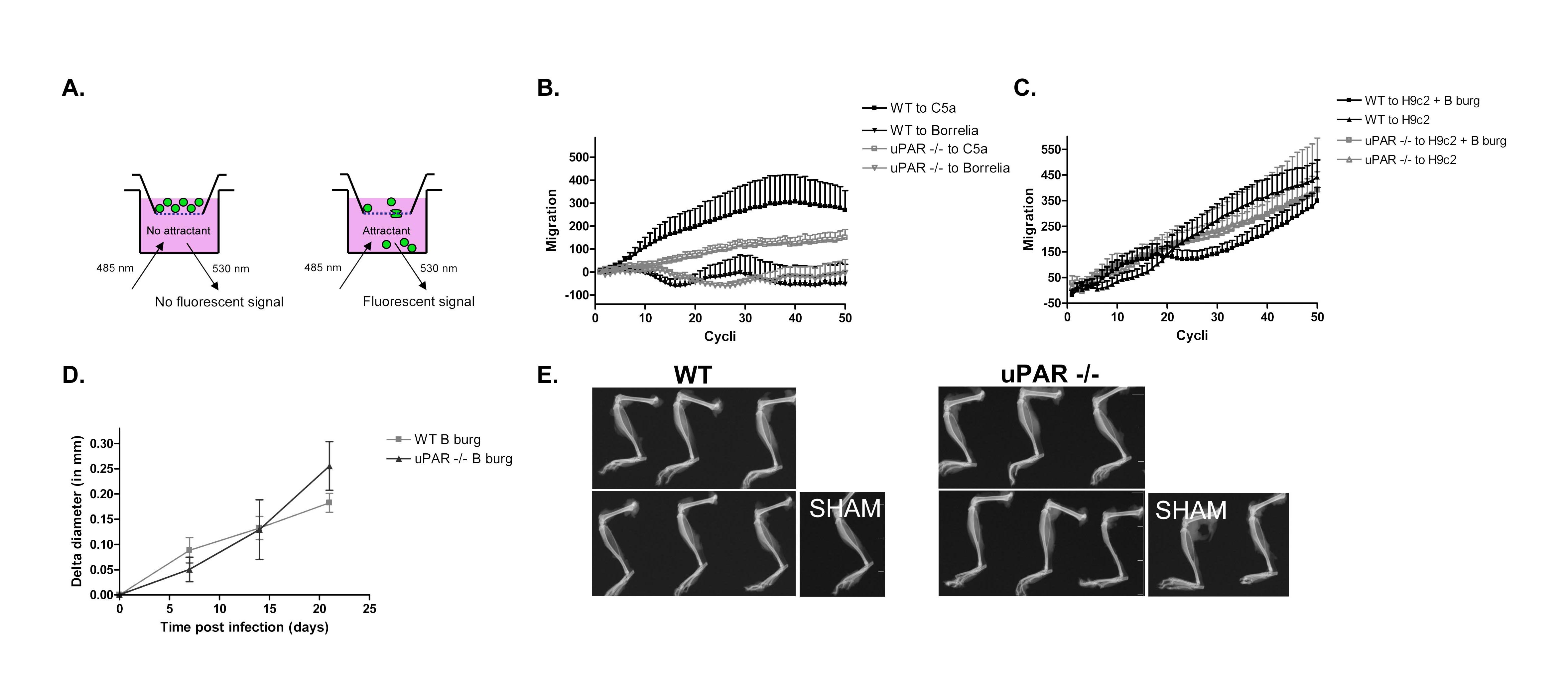

Supplement: Figure S5 — Migration and arthritis in WT and uPAR knock-out mice on a B. burgdorferi susceptible genetic background. (A, B and C) Urokinase receptor deficient macrophages from mice on the mixed genetic background can migrate to cardiogenic stimuli just as well as macrophages from WT littermate controls. Migration of CellTracker Green labeled WT or uPAR deficient macrophages towards several chemotactic stimuli was investigated in vitro (A). As chemotactic stimuli we used B. burgdorferi or activated complement factor 5 (C5a) (B) and supernatant from the cardiomyoblastic rodent cell line H9c2 stimulated with B. burgdorferi or control medium for 16 hours prior to experimentation (C). All conditions were tested in duplo, in serum free DMEM medium without the addition of antibiotics, and migration was corrected for the no-attractant control. Graphs represent the mean of three independent experiments±SEM. The fluorescent signal in the lower chamber (indicative of migration) was measured in real time every two minutes (cycli). (D and E) Only edema, no arthritis in B. burgdorferi infected uPAR knock-out mice (n = 7) and B. burgdorferi infected WT littermate controls (n = 8). Ankle swelling was measured using a microcaliper during the course of B. burgdorferi infection (D). In this particular experiment mice were monitored for three weeks. Post mortem, but before decalcification, radiological examination of the right hindlimb was performed (E). No differences between sham inoculated and B. burgdorferi infected animals were observed. A p-value<0,05 was considered statistically significant. * indicating p<0,05. (0.47 MB JPG) [file ppat.1000447.s005.jpg]
